# Primed Tactile Stimulus Processing during Sleep

**DOI:** 10.3390/life13112216

**Published:** 2023-11-16

**Authors:** Gonca Inanc, Murat Ozgoren

**Affiliations:** 1 Department of Biophysics, Faculty of Medicine, Near East University, 99138 Nicosia, Cyprus; 2 Department of Neuroscience, Post Graduate Institute, Near East University, 99138 Nicosia, Cyprus; 3 Brain and Conscious States Research Center, Near East University, 99138 Nicosia, Cyprus

**Keywords:** primed stimulus, sleep cognition, electrophysiology, non-painful tactile stimuli, P50, N300

## Abstract

The aim was to investigate how the primed and unprimed non-painful tactile stimuli during sleep would be processed. A total of 22 healthy subjects (19.55 ± 1.10 years) were randomly divided into two groups. The same stimuli were applied to both groups, but the study group (SG) received them twice (daytime and sleep), whereas the control group (CG) received them only during sleep. A 40-channel PSG and a pneumatic tactile stimulator unit were used. Evoked potential components of the C_Z_ electrode were examined in four sleep stages (N1, N2, N3, and REM). The Mann–Whitney U test was used for group comparison, and the Wilcoxon test was used for in-group evaluations. The P50 and N300 response components were observed in all sleep stages in both groups. P50 decreased as sleep deepened in the SG. The N300 increased as sleep deepened and started to decrease again in the REM stage. Moreover, in N1, the amplitudes of P200-N300 and N300-P450 in the SG were significantly greater than those in the CG. The fact that P50 was observed even in N3 indicates that bottom-up sensory processing continues during sleep. Moreover, the central processing of primed and unprimed stimuli exhibited dynamic differences. Furthermore, an increase in N300 amplitude suggests suppressive processes to facilitate and maintain sleep.

## 1. Introduction

Sleep is an important and complex process in terms of the biological balance of mammals. Changes in brain dynamics during sleep show that cognitive information is processed during sleep as well as in wakefulness, and that the brain is not completely disconnected from the external environment during sleep. It has been shown that external stimuli applied during sleep have different effects, and even some cognitive processes occur during sleep [1,2,3]. In addition, it is known that cognitive processes such as memory are also positively affected by sleep and have an important role in consolidation [4,5,6,7,8].

Sleep is not only a complex process but also a variable one, as the sleep process consists of different stages. Basically, according to the classic view, sleep consists of two main stages. One of these is the Rapid Eye Movement (REM) stage, and the other is the Non-Rapid Eye Movement (Non-REM or NREM) stage. NREM consists of three gradually deepening phases. These are N1, N2, and N3. The polysomnography (PSG) system can be used to simultaneously record and analyze many different physiological variables during sleep. The basic recording components of the PSG are electroencephalography (EEG), electrooculography (EOG), and electromyography (EMG). Basic recording components of the PSG are used to determine sleep stages. Besides the basic recording components, there are also components used to record respiratory parameters, muscle activity, blood pressure, snoring, and body position. These components are then used to describe sleep physiology and disorders.

Evoked potentials are often used in sleep research instead of other approaches. The main reason for this is the inability to give cognitive tasks to individuals during sleep and the inability to receive cognitive or behavioral responses from individuals. Different stimuli, such as auditory, visual, tactile, smell, and taste, can be used in evoked potentials. The electrical activity of the brain against these external stimuli is observed as the voltage deviations that can be recorded on the skin resulting from the aggregated postsynaptic potentials of large groups of nerve cells and can be recorded with the electroencephalography (EEG) system. The recorded neural responses can be determined with signal processing methods, and the amplitude and latency of consecutive peaks provide information about the information processing of different sensory and cognitive events. These successive peaks are usually named after the period in which they occur, like the P50 and N300 peaks mentioned in this research. The peak that occurs in a positive direction between 50 and 120 ms after the stimulus is called P50. This peak is a positive component related to sensory processing [9]. The negative peak that occurs approximately 250 to 400 ms after the stimulus is called N300. This peak is not seen during wakefulness and is a sleep-specific component. The N300 response is thought to be related to the semantic content of the stimulus [2]. Regarding research on the response of the human brain in the somatosensory area, painful tactile stimuli such as electrical or laser stimuli have generally been used [10,11,12,13]. However, it is known that the components of the evoked potentials that occur in response to painful tactile stimuli are different in sleep processes [14,15]. The number of studies examining the evoked potential of non-painful, simple tactile stimuli during sleep in adults is very limited [2,16,17]. In these studies, stimuli were applied to individuals only during sleep or just before sleep. There is limited information with respect to sleep cognition and the sleep sensory/cognitive processes against the priming of simple external real-life stimulations. Accordingly, in the present study, it was aimed to examine the evoked potentials of primed and unprimed non-painful tactile stimuli during sleep stages.

## 2. Materials and Methods

**Figure 1:**
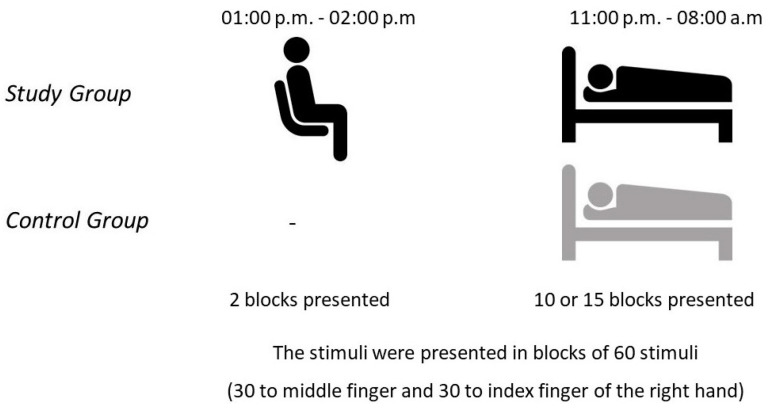
The experimental design and the recording procedure for the study and control groups.

A total of 22 healthy subjects participated in the study (mean age: 19.55 ± 1.10 years). Subjects in the study were randomly divided into two groups: primed and unprimed. Non-painful tactile stimuli were applied to the primed group during the day and the same non-painful tactile stimuli were applied to the same subjects the same night while the subjects were sleeping in the laboratory (study group/SG, N = 11, mean age: 19.27 ± 0.90 years) (Table 1). In the unprimed group, non-painful tactile stimuli were applied only during the night (control group/CG, N = 11, mean age: 19.82 ± 1.25 years) (Figure 1).

The study was approved by the Non-Interventional Ethics Committee on 3 March 2016 (Decision No: 2016/06-03). The study was carried out in accordance with the rules stated in the Declaration of Helsinki. Before the recording, several forms and scales were applied to the subjects after their consent was obtained. These were: personal information form, psychological symptom screening test (SCL-90R), state anxiety scale (STAI-TX1), Epworth Sleep Scale, Pittsburgh Sleep Quality Index, and Edinburgh Handedness Inventory. The personal information form was used to determine whether some of the participants’ personal information and habits, such as medication, substance, and coffee and alcohol use, could affect the registration used on the day of registration. STAI-TX1 and SCL-90R were used to determine whether subjects had symptoms of anxiety or any psychiatric illness. The Pittsburgh Sleep Quality Index (PSQI) was used to evaluate participants’ sleep quality, and the Epworth Sleep Scale was used to evaluate daytime sleepiness. The Edinburgh Handedness Inventory was used to determine participants’ hand preferences.

Volunteer subjects slept in a special isolated room, isolated from electromagnetic waves and sound. Communication with the subjects was provided by the sound system between the two rooms, and the room was monitored with a camera during the recording with the knowledge and permission of the subjects. Stimuli during wakefulness were applied to the participants in the SG in a comfortable chair in the same room.

In addition to the 40-channel polysomnography system (PSG) consisting of electroencephalography (EEG), electromyography (EMG), and electrooculography (EOG), an Embedded Microcontroller Stimulation Unit (EMISU) [18], a pneumatic stimulator unit (Somatosensory Stimulus Generator 4-D Neuroimaging), and a video recording system were used. 

During EEG recording, a Quick Cap of appropriate size was utilized. Electro-Gel was used to ensure conductivity between the electrodes and the scalp (Electro-Gel, Electro-Cap International, Inc., Eaton, OH, USA). The earlobes were used for EEG referencing ([A1 + A2]/2). During the EEG recordings, the impedances of the electrodes were kept at approximately 5 kOhm, and a continuous EEG recording was taken with a sample rate of 1 kHz through a NuAmps (Neuroscan Labs, Charlotte, NC, USA) recording system. 

The EOG system was used to record eye movements. EOG electrodes were placed 1 cm below the canthus of the right eye and 1 cm above the outer canthus of the left eye. For EMG recordings, electrodes were placed above and below the mandible over the mental and submental muscles of the jaw.

The pneumatic stimulus unit was used for non-painful tactile stimuli. In the recordings, non-painful tactile stimuli were applied to the subjects by means of clips with an 8–9 mm movable synthetic membrane on the surface contacting the finger pulp. The pneumatic stimulator unit is a device that transmits and draws dry air from the dry air tube to the cables leading to the clips, according to the experimental pattern prepared in the MATLAB software (MATLAB R2020b) environment. The membranes inside the clips move with the dry air coming into the clips, creating a painless touch effect on the fingertips of the volunteer participants. One type of pressure stimulus was applied to the index and middle fingers of the subjects’ right hands, after 30 stimuli were applied to one finger one after the other, and the other 30 stimuli were applied to the other finger one after the other. 

Blocks consisting of 60 stimuli and lasting an average of 8–10 min were applied to the participants. While 2 blocks were applied to the study group (SG) during the awake period, between 10 and 15 blocks were applied to both the SG and control group (CG) during the night during sleep (Figure 1).

The order of arrival of the applied stimuli on the fingers of the subjects and the time between the stimuli were randomized. The interstimulus interval (ISI) was determined as 3–3.5 s.

EEG analyses were performed after recording. Sleep stages were evaluated according to the American Academy of Sleep Medicine (AASM) scoring system [19]. The basic PSG components, EEG, EOG, and EMG, were used to determine sleep stages. The recordings were examined one by one every 30 s, and the sleep stages were determined. In this study, EEG data were divided into four groups: stage 1 (N1), stage 2 (N2), stage 3 (N3), and the Rapid Eye Movement (REM) stage. For each stimulus at each stage, sweeps covering the period of 1000 ms before the stimulus and 2000 ms after the stimulus were created. Signals with an amplitude higher than ±100 μV in the EOG channel and signals containing noise were removed. The remaining sweeps were baseline-corrected with a digital 0.5–48 Hz band-pass filter (Neuroscan 4.5). The separate mean files of all participants for N1, N2, N3, and the REM stage were created. In the research, the time at which the response components appeared after the stimulus was applied was called latency, and the voltage values of the response components were called amplitude. The latencies of the response components were measured in milliseconds and the amplitudes in microvolts were consecutively measured and evaluated for P50, N300, P200-N300, and N300-P450. In the SG after the stimulus was applied, the positive peak between 56–152 ms was evaluated as P50, the positive peak between 146–254 ms was evaluated as P200, the negative peak between 262–418 ms was evaluated as N300, and the positive peak between 404–636 ms was evaluated as P450. In the CG after the stimulus was applied, the positive peak between 32–180 ms was evaluated as P50, the positive peak between 156–290 ms was evaluated as P200, the negative peak between 280–410 ms was evaluated as N300, and the positive peak between 362–660 ms was evaluated as P450.

**Figure 2:**
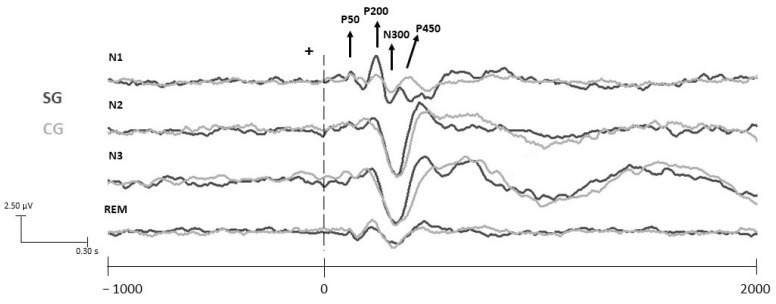
Brain responses to non-painful tactile stimuli in study and control groups during sleep stages (N1, N2, N3, and the REM stage) at C_Z_ electrode site. Stimulus onset time was marked at the “0” point with a vertical dashed line. Horizontal axis represents time axis with a 1000 ms pre-stim and 2000 ms post-stim area. Vertical axis represents amplitude values in microvolts; the bottom side has negative values and the top side has positive values. Dark gray line represents study group (SG, N = 11), and light gray line represents control group (CG, N = 11).

The P200-N300 distance was calculated with the amplitude difference between the P200 and N300 peaks, and the N300-P450 peak was calculated with the amplitude difference between the N300 and P450 peaks (Figure 2).

For the statistical analysis, a two-way ANOVA was used for two-group comparisons, and the Wilcoxon test was used for within-group evaluations in the SPSS 22 program (IBM SPSS Statistics 22, IBM Corp., New York, NY, USA). A value of *p* < 0.05 was accepted for statistical significance.

## 3. Results

In the current study, no significant difference was found between the SG and the CG in terms of age and gender. Thus, the two groups were regarded as similar. It was observed that the P50 and N300 response components appeared in all electrode regions after the application of tactile stimuli in both the SG and CG (Figure 2). For the sake of simplicity, only the findings of the C_Z_ (central) electrode are presented below.

### 3.1. P50 Component

After the stimulus was applied to the SG, the latencies of the P50 response component were measured as 125.09 ± 18.58 ms in N1, 116.18 ± 21.88 ms in N2, 100.00 ± 34.22 ms in N3, and 107.82 ± 22.48 ms in the REM stage. In the CG, the average latencies of the P50 response component were measured as 118.18 ± 20.29 ms in N1, 101.27 ± 33.86 ms in N2, 101.45 ± 39.21 ms in N3, and 100.55 ± 22.61 ms in the REM stage. There were no significant differences observed between the stages in the latencies of P50 in the SG. It was observed that the P50 response component emerged significantly earlier in the REM stage than in N1 for the CG (*p* = 0.012). 

**Figure 3:**
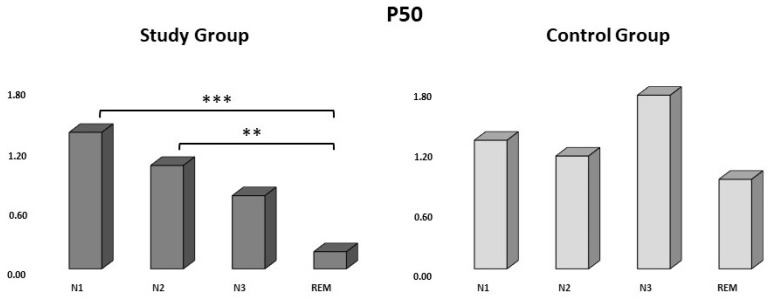
The average amplitude of P50 responses to the study and control groups during N1, N2, N3, and REM sleep (recorded from C_Z_). Y-axis shows the amplitude of the P50 component in microvolts. Dark gray bars represent the study (primed) group (N = 11), and light gray bars represent the control group (N = 1) (** *p* ≤ 0.01, *** *p* ≤ 0.001).

The P50 amplitude was 1.36 ± 0.82 µV in N1, a mean of 1.03 ± 0.67 µV in N2, 0.73 ± 0.73 µV in N3, and 0.17 ± 0.49 µV in the REM stage in the SG (Table 2). The amplitude of the P50 response component occurring in the REM stage in the study group was found to be significantly smaller than the P50 response components occurring in N1 and N2 (*p* = 0.004 and *p* = 0.010, respectively) (Figure 3). In the control group, the mean amplitude of P50 was measured as 1.28 ± 0.82 µV in N1, 1.13 ± 1.14 µV in N2, 1.73 ± 1.48 µV in N3, and 0.89 ± 1.01 µV in the REM stage (Table 2).

For the P50 component, the group (study or control) × sleep stages (N1, N2, N3, and the REM stage) ANOVA was conducted. The results revealed a significant main effect on the group, such that those of the CG were significantly greater than those of the SG (F (1,80) = 4.707, *p* = 0.033). Furthermore, there was a significant main effect of the sleep stages for N3 and the REM stage (F (3,80) = 3.117 *p* = 0.031), but there was no significant group–sleep-stage interaction (F (3,80) = 1.629, *p* = 0.189).

### 3.2. N300 Component

In the SG, the latency of the N300 response component was measured as 347.82 ± 48.84 ms in N1, 327.64 ± 23.27 ms in N2, 329.45 ± 28.10 ms in N3, and 312.18 ± 21.72 ms in the REM stage, after the stimulus was applied. In the CG, the mean latency of the N300 response component was 328.73 ± 37.30 ms in N1, 334.73 ± 26.25 ms in N2, 341.45 ± 40.63 ms in N3, and 322.00 ± 16.97 ms in the REM stage. No significant difference was observed between the sleep stages in terms of latency, neither in the SG nor CG.

**Figure 4:**
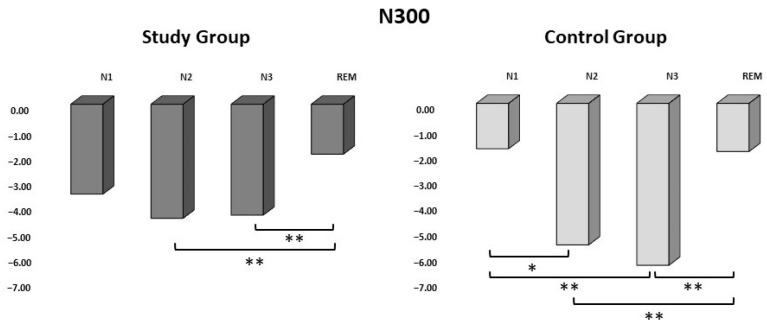
The average amplitude of N300 responses to the study and control groups during N1, N2, N3, and REM sleep represented through the C_Z_ electrode. Y-axis shows the amplitude of the N300 component in microvolts. Dark gray bars represent the study group (N = 11), and light gray bars represent the control group (N = 11) (* *p* < 0.05, ** *p* ≤ 0.01).

In the SG, the mean amplitude of N300 was measured as −3.52 ± 2.17 µV in N1, −4.47 ± 2.65 µV in N2, −4.34 ± 2.82 µV in N3, and −1.96 ± 1.53 µV in the REM stage (Table 3). The amplitude of the N300 response component to tactile stimuli in the SG was found to be significantly greater in the REM stage than in N2 and N3 (*p* = 0.003; *p* = 0.010) (Figure 4).

In the CG, the mean amplitude of N300 was measured as −1.77 ± 0.95 µV in N1, −5.52 ± 2.76 µV in N2, −6.31 ± 1.98 µV in N3, and −1.88 ± 1.09 µV in the REM stage (Table 3). In the CG, the amplitude of N300 to tactile stimuli was found to be significantly greater in N3 than in N1 and the REM stage. Moreover, the N300 amplitude of N2 was significantly greater than that of N1 or the REM stage (*p* = 0.008; *p* = 0.003; *p* = 0.021; *p* = 0.003) (Figure 4).

In the SG, P200-N300 potentials were measured as 6.30 ± 4.07 µV in N1, 5.89 ± 3.54 µV in N2, 6.18 ± 3.47 µV in N3, and 2.94 ± 2.42 µV in the REM stage, while in the CG, P200-N300 potentials were measured as 1.59 ± 1.08 µV in N1, 7.41 ± 3.71 µV in N2, 8.04 ± 3.13 µV in N3, and 3.66 ± 1.49 µV in the REM stage.

In the SG, N300-P450 potentials were measured as 4.68 ± 2.33 µV in N1, 7.70 ± 4.30 µV in N2, 7.13 ± 5.08 µV in N3, and 3.39 ± 2.50 µV in the REM stage. In the CG, N300-P450 potentials were measured as 3.45 ± 1.93 µV in N1, 8.25 ± 4.92 µV in N2, 8.35 ± 3.40 µV in N3, and 2.80 ± 1.46 µV in the REM stage.

For P200-N300, the group (study or control) x sleep stages (N1, N2, N3, and the REM stage) ANOVA was conducted. Results revealed a significant main effect of group and sleep stages ((F (1,80) = 21.379 *p* = 0.000), (F (3,80) = 45.332 *p* = 0.000)). Group-stage interaction significance was found for P200-N300 (F (3,80) = 6.449 *p* = 0.001). In post hoc analyses, the response in the study group was found to be significantly greater than that in the control group in N1 (*p* ≤ 0.000). For N300-P450, the group and sleep stage main effects were significant ((F (1,80) = 7.368 *p* = 0.008), (F (3,80) = 31.529 *p* = 0.000)). Group-stage interaction significance was found for N300-P450 (F (3,80) = 5.091 *p* = 0.003). In post hoc analyses, the response in the study group was found to be significantly greater than that in the control group in N1 (*p* ≤ 0.000).

## 4. Discussion

The current study clearly presents that the P50 and N300 non-painful tactile brain response components occurred in both the primed and unprimed groups. Furthermore, the components differed in terms of latencies and amplitudes in different periods of sleep in both groups.

In the literature, studies examining brain responses to non-painful tactile stimuli during sleep are very limited [2,16,17]. Oniz et al. presented a report of non-painful tactile stimuli that were applied to the participants during sleep or just before sleep without priming [2].

In contrast, during the present study, tactile stimuli were applied to the subjects both while awake and asleep (study group) and only during sleep. Thus, the brain responses of the subjects to tactile stimuli, both primed and unprimed, during sleep were evaluated.

### 4.1. P50 Component

In adults, studies in which tactile stimuli were applied during wakefulness have reported that the P50 response component appeared on average 50 to 120 ms after the stimulus [19]. In the present study, although non-painful tactile stimuli were applied during sleep, the duration of the P50 response component in both groups was consistent with the literature.

Lught et al. reported that the response components, which usually occur after 50 ms, were significantly affected by sleep [20]. There is some debate about the variation of the response component, which peaks at an average of 50 ms and is also called P1 in the literature, during sleep. While some studies reported that the amplitude of this response component decreased, others reported that it increased [21,22,23].

It has been stated that the amplitude of the early evoked potential response component P1 decreases as sleep deepens and its latency gets longer [22,24].

In a study using auditory stimuli in the literature, it was reported that the amplitude of the response component that occurred 15 to 50 ms after the stimulus decreased from wakefulness to slow-wave sleep and could exceed wakefulness values in REM sleep [25].

In the present study, it was observed that the amplitude of the P50 response component decreased as sleep deepened in the SG and this decrease continued in REM sleep, but this was not the case in the CG. When the latency of the response component was examined, it was found that P50 appeared earlier in REM sleep than in N1 only in the CG. As also stated in the literature, the decrease in the amplitude of the P50 response component as sleep deepens was observed only in the SG in this study. Since the participants were primed with stimuli, the amplitude decreased as the sleep deepened. This finding suggested that the processing of external stimuli in the brain decreased as the sleep deepened. In the control group, this process continued during the sleep process as the participants did not know (unprimed) or have prior experience with these specific stimuli. 

In the literature, it has been stated that the early components of sleep-evoked potentials generally contain sensory information, while the later components are more sleep-protective [26]. In both groups, the P50 response component was observed even in N3, the deepest sleep, indicating that sensory processing continued during sleep. However, we observed dynamic interplay in sensory processing between primed and unprimed stimuli.

### 4.2. N300 Component

In the present study, it was found that the amplitude of the N300 response component increased as sleep deepened (especially in N2 and N3) and started to decrease again in the REM stage in both groups. Additionally, in N1, P200-N300 and N300-P450 potentials were greater in the SG.

The N300 response component is a negative component that occurs 250–400 ms after the stimulus. This component begins to appear during the transition from a drowsy state to sleep. Like the N1 component, it was reported that the N2 component also shrunk during sleep and the N350 component emerged [27]. In the opinion of Crowley and Colrain (2004), it has been reported that the N350 component is one of the largest response components that can be recorded during sleep, and this can be explained by the intense firing of a large neuron ensemble [27,28]. It has been stated that a component of this magnitude can possibly be explained by the synchronized firing of large numbers of cortical cells; therefore, their presence, delay, and, most importantly, amplitudes functionally measure the integrity of the central nervous system as a whole. It has been claimed that white matter deterioration leads to decreased synchronization and gray matter dysfunction can affect the amplitude of the potential as a result of the sum of synchronized activity. The N300 component may be associated with sleep facilitation and protection processes [29]. The results of the current study were thus similar to those of these studies. 

Kállai et al. (2003) reported that N350, which peaks during sleep, did not have information transfer properties, but the bursts in the burst-stall firing mode during sleep were only one of the excitatory waves, and its negativity was the neurological counterpart of neuronal activities [30,31]. Unlike Kallai, in our study, N300 was measured. It remains in the scope of further analysis whether they fall under the same component. 

It was reported that the N300 response component may have been related to the semantic content of the stimulus and the increase in amplitude may have been related to the waking processes [32]. This current study differed from the opinion of N300 being related to sleep processes. 

In another study, it was reported that the increase in the amplitude of this response component reflected the suppressive processes, not the awakening processes [33]. Yang et al. stated that the amplitude increase seen in the N300 response component was proportional to the increase in the delta sleep rate, and this component may have been a component related to the need for slow-wave sleep [3]. 

Similarly, in the present study, the increase in the amplitude of the N300 response component as sleep deepened in both groups indicated that this response component reflected suppressive processes to facilitate and protect sleep.

The fact that P200-N300 and N300-P450, which make up the N300 response component, were greater in the study group than in the control group only in N1 indicated that this response component exerted greater suppression to induce sleep against known stimuli.

The current study did not refer to laterality and regional activations. Moreover, the functional representation scope was not addressed. A further research scope should handle these aspects. 

## 5. Conclusions

The introduction (priming) of the tactile stimuli before the night of sleep resulted in a number of changes in the way the brain processes external stimuli. Hence, the top-down and bottom-up processes continued in different sleep stages. This report hints at the dynamics of somatosensory processing in conjunction with sleep cognition as a first study. The results could be used in further clinical (e.g., sensory malfunctioning) and sensory research for understanding the sleeping brain and brain processes in general. Finally, further cognitive processes could be explored in an objective setup during the sleep–daytime cycles.

## Figures and Tables

**Table 1:** Gender and age distribution of participants divided into two groups (study and control), N = 22.

	Gender	N	Mean Age
Study Group (SG), N = 11	Female	3	19.33 ± 1.53
	Male	8	19.25 ± 0.71
Control Group (CG), N = 11	Female	4	19.00 ± 1.41
	Male	7	20.29 ± 0.95

**Table 2:** The mean, standard error (SEM), standard deviation (SD), and minimum (Min) and maximum (Max) amplitude values of the P50 components in the study group (SG) and control group (CG) are shown.

P50								
	N1		N2		N3		REM	
	SG	CG	SG	CG	SG	CG	SG	CG
Mean	1.36	1.28	1.03	1.13	0.73	1.73	0.17	0.89
SEM.	0.25	0.25	0.20	0.34	0.22	0.45	0.15	0.30
SD.	0.82	0.82	0.67	1.14	0.73	1.48	0.49	1.01
Min.	−0.08	−0.35	0.22	−0.62	−0.32	−1.11	−0.36	−1.12
Max.	3.11	2.26	2.66	2.91	1.72	4.12	1.06	2.22

**Table 3:** The mean, standard error (SEM), standard deviation (SD), and minimum (Min) and maximum (Max) amplitude values of the N300 components in the study group (SG) and control group (CG) are shown.

N300								
	N1		N2		N3		REM	
	SG	CG	SG	CG	SG	CG	SG	CG
Mean	−3.52	−1.77	−4.47	−5.52	−4.35	−6.31	−1.96	−1.88
SEM.	0.65	0.29	0.80	0.83	0.85	0.60	0.46	0.33
SD.	2.17	0.95	2.65	2.76	2.82	1.98	1.53	1.09
Min.	−8.02	−3.49	−10.39	−11.73	−9.43	−9.91	−6.19	−3.18
Max.	−0.80	−0.82	−1.33	−2.94	−0.61	−2.86	−0.74	−0.03

## Data Availability

The data presented in this study are available on request from the corresponding author. The data are not publicly available because the data collection is ongoing.

## References

[1] Özgören M., Kocaaslan S., Önız A. (2008). Analysis of Non-REM Sleep Staging with Electroencephalography Bispectral Index. Sleep Biol. Rhythm..

[2] Oniz A., Inanc G., Guducu C., Ozgoren M. (2016). Brain Responsiveness to Non-Painful Tactile Stimuli Prior and during Sleep. Sleep Biol. Rhythm..

[3] Yang C.-M., Wu C.-S. (2007). The Effects of Sleep Stages and Time of Night on NREM Sleep ERPs. Int. J. Psychophysiol..

[4] Watson B.O., Buzsáki G. (2015). Sleep, Memory & Brain Rhythms. Daedalus.

[5] Rasch B., Born J. (2013). About Sleep’s Role in Memory. Physiol. Rev..

[6] Walker M.P., Stickgold R. (2004). Sleep-Dependent Learning and Memory Consolidation. Neuron.

[7] Oniz A., Inanc G., Guducu C., Ozgoren M. (2015). Explicit and Implicit Memory during Sleep. Sleep Biol. Rhythm..

[8] Uji M., Tamaki M. (2023). Sleep, Learning, and Memory in Human Research Using Noninvasive Neuroimaging Techniques. Neurosci. Res..

[9] Öniz A., Güdücü Ç., Bayazıt O., Özgören M. (2009). Dokunsal olay ilişkili yanıtlar ışığında öğrenme sürecinin irdelenmesi. The Assessment of Learning through the Scope of Somatosensory Event Related Potentials. Meandros Med. Dent. J..

[10] Kakigi R., Hoshiyama M., Shimojo M., Naka D., Yamasaki H., Watanabe S., Xiang J., Maeda K., Lam K., Itomi K. (2000). The Somatosensory Evoked Magnetic Fields. Progress Neurobiol..

[11] Tran T.D., Inui K., Hoshiyama M., Lam K., Kakigi R. (2002). Conduction Velocity of the Spinothalamic Tract Following CO_2_ Laser Stimulation of C-Fibers in Humans. Pain.

[12] Yamasaki H., Kakigi R., Watanabe S., Naka D. (1999). Effects of Distraction on Pain Perception: Magneto- and Electro-Encephalographic Studies. Brain Res. Cogn. Brain Res..

[13] Nakata H., Aoki M., Sakamoto K. (2017). Effects of Mastication on Human Somatosensory Processing: A Study Using Somatosensory-Evoked Potentials. Neurosci. Res..

[14] Yamada T., Kameyama S., Fuchigami Y., Nakazumi Y., Dickins Q.S., Kimura J. (1988). Changes of Short Latency Somatosensory Evoked Potential in Sleep. Electroencephalogr. Clin. Neurophysiol..

[15] Nakano S., Tsuji S., Matsunaga K., Murai Y. (1995). Effect of Sleep Stage on Somatosensory Evoked Potentials by Median Nerve Stimulation. Electroencephalogr. Clin. Neurophysiol..

[16] Inanç G., Özgören M., Öniz A. (2021). Sensory Brain Responses and Lateralization in Nonpainful Tactile Stimuli during Sleep. Neurol. Sci. Neurophysiol..

[17] Inanc G., Ozgoren M., Oniz A. (2022). Investigation of Non-Painful Tactile Stimuli in Sleep: Amplitude and Frequency Analysis. Cyprus J. Med. Sci..

[18] Ozgoren M., Erdogan U., Bayazit O., Taslica S., Oniz A. (2009). Brain Asymmetry Measurement Using EMISU (Embedded Interactive Stimulation Unit) in Applied Brain Biophysics. Comput. Biol. Med..

[19] Berry R.B., Brooks R., Gamaldo C., Harding S.M., Lloyd R.M., Quan S.F., Troester M.T., Vaughn B.V. (2017). AASM Scoring Manual Updates for 2017 (Version 2.4). J. Clin. Sleep Med..

[20] De Lugt D.R., Loewy D.H., Campbell K.B. (1996). The Effect of Sleep Onset on Event Related Potentials with Rapid Rates of Stimulus Presentation. Electroencephalogr. Clin. Neurophysiol..

[21] Osterhammel P.A., Shallop J.K., Terkildsen K. (1985). The Effect of Sleep on the Auditory Brainstem Response (ABR) and the Middle Latency Response (MLR). Scand. Audiol..

[22] Erwin R., Buchwald J.S. (1986). Midlatency Auditory Evoked Responses: Differential Effects of Sleep in the Human. Electroencephalogr. Clin. Neurophysiol..

[23] Campbell S.S., Murphy P.J. (2007). The Nature of Spontaneous Sleep across Adulthood. J. Sleep Res..

[24] Kevanishvili Z.S., Von Specht H. (1979). Human Slow Auditory Evoked Potentials during Natural and Drug-Induced Sleep. Electroencephalogr. Clin. Neurophysiol..

[25] Picton T.W., Hillyard S.A. (1974). Human Auditory Evoked Potentials. II: Effects of Attention. Electroencephalogr. Clin. Neurophysiol..

[26] Steriade M., McCormick D.A., Sejnowski T.J. (1993). Thalamocortical Oscillations in the Sleeping and Aroused Brain. Science.

[27] Coenen A. (2012). Modelling of Auditory Evoked Potentials of Human Sleep-Wake States. Int. J. Psychophysiol..

[28] Crowley K.E., Colrain I.M. (2004). A Review of the Evidence for P2 Being an Independent Component Process: Age, Sleep and Modality. Clin. Neurophysiol..

[29] Colrain I.M., Campbell K.B. (2007). The Use of Evoked Potentials in Sleep Research. Sleep Med. Rev..

[30] Kállai I., Harsh J., Voss U. (2003). Attention to External Stimuli during Wakefulness and Sleep: Evoked 40-Hz Response and N350. Psychophysiology.

[31] Coenen A.M. (1995). Neuronal Activities Underlying the Electroencephalogram and Evoked Potentials of Sleeping and Waking: Implications for Information Processing. Neurosci. Biobehav. Rev..

[32] Bastuji H., García-Larrea L. (1999). Evoked Potentials as a Tool for the Investigation of Human Sleep. Sleep Med. Rev..

[33] Peszka J., Harsh J. (2002). Effect of Sleep Deprivation on NREM Sleep ERPs and Related Activity at Sleep Onset. Int. J. Psychophysiol..

